# The prognostic value of long noncoding RNA SNHG16 on clinical outcomes in human cancers: a systematic review and meta-analysis

**DOI:** 10.1186/s12935-019-0971-2

**Published:** 2019-10-11

**Authors:** Chenghao Zhang, Xiaolei Ren, Jieyu He, Wanchun Wang, Chao Tu, Zhihong Li

**Affiliations:** 1 Department of Orthopedics, The Second Xiangya Hospital, Central South University, No 139 Middle Renmin Road, Changsha, 410011 Hunan China; 2 Hunan Key Laboratory of Tumor Models and Individualized Medicine, The Second Xiangya Hospital, Central South University, Changsha, Hunan China; 3 Department of Geriatrics, The Second Xiangya Hospital, Central South University, Changsha, Hunan China; 4 University of Texas Health Science Center at San Antonio, San Antonio, TX USA

**Keywords:** LncRNA, Cancer, Sarcoma, SNHG16, Prognosis, Metastasis

## Abstract

**Background:**

Cancer has been a worldwide health problem with a high risk of morbidity and mortality, however ideal biomarkers for effective screening and diagnosis of cancer patients are still lacking. Small nucleolar RNA host gene 16 (SNHG16) is newly identified lncRNA with abnormal expression in several human malignancies. However, its prognostic value remains controversial. This meta-analysis aimed to synthesize available data to clarify the association between SNHG16 expression levels and clinical prognosis value in multiple cancers.

**Methods:**

Extensive literature retrieval was conducted to identify eligible studies, and data regarding SNHG16 expression levels on survival outcomes and clinicopathological features were extracted and pooled for calculation of the hazard ratios (HRs) or odds ratios (ORs) with 95% confidence intervals (CIs). Forest plots were applied to show the association between SNHG16 expression and survival prognosis. Additionally, The Cancer Genome Atlas (TCGA) dataset was screened and extracted for validation of the results in this meta-analysis.

**Results:**

A total of eight studies comprising 568 patients were included in the final meta-analysis according to the inclusion and exclusion criteria. In the pooled analysis, high SNHG16 expression significantly predicted worse overall survival (OS) in various cancers (HR = 1.87, 95% CI 1.54–2.26, P < 0.001), and recurrence-free survival (RFS) in bladder cancer (HR = 1.68, 95% CI 1.01–2.79, P = 0.045). Meanwhile, stratified analyses revealed that the survival analysis method, tumor type, sample size, and cut-off value did not alter the predictive value of SNHG16 for OS in cancer patients. In addition, compared to the low SNHG16 expression group, patients with high SNHG16 expression were more prone to worse clinicopathological features, such as larger tumor size, advanced clinical stage, lymph node metastasis (LNM) and distant metastasis (DM). Exploration of TCGA dataset further validated that the upregulated SNHG16 expression predicted unfavorable OS and disease-free survival (DFS) in cancer patients.

**Conclusions:**

The present study implicated that aberrant expression of lncRNA SNHG16 was strongly associated with clinical survival outcomes in various cancers, and therefore might serve as a promising biomarker for predicting prognosis of human cancers.

## Background

Nowadays, cancer has become one of the most prevalent causes of mortality worldwide [1]. Over the past century, there has been a dramatic improvement in modern treatments for cancer including surgery, adjuvant therapy and supportive therapy [2–4]. Despite this, the patients’ survival rate are still unsatisfied and quality of life remains largely to be improved, especially for those with advanced clinical stage or metastasis [5]. It has been well established that early diagnosis and treatment of cancer could greatly reduce its mortality. However, the insufficiency of suitable biomarkers presents a major obstacle to this issue [6]. Consequently, there is an urgent need to find new biological targets in the carcinogenesis of tumors.

LncRNAs is a class of RNAs with a length of more than 200 nucleotides (nt) [7]. Previous evidences suggest that lncRNAs can regulate gene expression at all levels-transcriptional, translational, and post-translational-by interacting with DNA, RNA and protein [8], and subsequently accomplish a remarkable variety of biological processes [8, 9]. In recent years, an increasing number of lncRNAs have been revealed to be aberrantly expressed in human cancers [10]. Moreover, dysregulation of lncRNAs is significantly correlated with cancer cell proliferation, migration, metastasis and recurrence, implicating a crucial role of lncRNAs in regulation of carcinogenesis and cancer progression [7].

Small nucleolar RNA host gene 16 (SNHG16) is a recently identified lncRNA with abnormal expression in multiple cancers [11–14]. Increased expression of SNHG16 usually predicted poor prognosis in multiple cancers including osteosarcoma [15], bladder cancer [16–18], esophagus cancer [19], non-small cell lung cancer (NSCLC) [11], glioma [20, 21], oral squamous cell carcinoma [13], hepatocellular carcinoma (HCC) [22], breast cancer [23], and ovarian cancer [24]. In these cancers, high expression levels of SNHG16 were usually correlated with worse clinicopathological features, such as tumor size, clinical stage, lymph nodes metastasis (LNM), distant metastasis (DM), and drug resistance. For incidence, in HCC, SNHG16 predicted portal vein tumor thrombus and sorafenib resistance [25]. Moreover, SNHG16 may be also engaged in the pathogenesis and progression of cancers, including proliferation, migration and invasion [26]. Furthermore, emerging studies have demonstrated and emphasized the importance of lncRNA SNHG16 in regulation of cancer-related signaling pathways, including Wnt/β-catenin, PI3K/Akt, and JAK2/STAT3 pathway [21, 27–29]. Collectively, SNHG16 may serve as a risk factor and therapeutic target for several types of human malignancies. However, most researches evaluating the prognostic value of SNHG16 in cancer survivals are limited due to small sample size and the contentious outcomes in clinical settings. In this meta-analysis, we report, for the first time, the comprehensive role of SNHG16 expression in human pan-cancers, which may provide promising targets for the development of novel diagnostic and therapeutic strategies against cancers.

## Methods

### Publication search strategy

The present study was rigorously projected, reviewed and reported in accordance with the PRISMA checklist [30–32]. We searched numerous electronic databases, including MEDLINE, Web of Science, Scopus, the Cochrane Library, EmBase, and China National Knowledge Infrastructure (CNKI) for eligible studies from their inceptions up to Jan 1st, 2019. The following search items were used: “small nucleolar RNA host gene 16 OR SNHG16” AND “tumor OR cancer OR carcinoma OR sarcoma” with language limitation to English and Chinese. Additionally, the citation lists in these retrieved articles were manually searched for identification of other relevant studies to ensure sensitivity of the search strategy.

### Inclusion and exclusion criteria

Two investigators (CHZ and XLR) critically reviewed and assessed all eligible studies independently. Studies for inclusion should meet the following criteria: (a) SNHG16 expression level was examined in human cancer tissues and adjacent normal tissues; (b) patients were separated into high and low expression groups based on the cut-off value of SNHG16 expression; (c) sufficient data regarding association between SNHG16 expression and survival outcomes or clinicopathological features; and (d) estimated hazard ratios (HRs) or odds ratios (ORs) with corresponding 95% confidence intervals (CIs) for survival outcomes could be extracted directly or indirectly.

While those studies should be excluded if meet any one of the following criteria: (a) irrelevant to cancer and SNHG16; (b) focused on the molecular structure or functions of SNHG16 rather than its correlation with survival outcomes; (c) duplicate publications; (d) animal studies; and (e) publications without usable data, such as reviews, letters to the editor, and abstracts.

### Data extraction and quality control

The following information was extracted by two independent investigators (CHZ and XLR) from each selected study: Surname of first author, publication year, country of origin, tumor type, sample type and size, follow-up months, detection assay, clinical stage, metastasis, cut-off value, survival outcome including overall survival (OS), disease-free survival (DFS), progression-free survival (PFS), and recurrence-free survival (RFS). If data of interest were not accessible, we obtained the missing data by contacting the corresponding author of enrolled articles. If only Kaplan-Meier (K–M) curves were provided in some studies, we used the Engauge Digitizer (Version 4.1) to calculate the pooled HRs and 95% CIs through indirect extraction from the plots [33].

Since all studies included in our meta-analysis were cohort studies, Newcastle–Ottawa Scale (NOS) with score ranging from 0 to 9 was utilized to carry out the quality assessment by two investigators (JYH and WCW) [34]. Included studies with NOS score ≥ 7 were considered of high methodological quality.

### Validation by reviewing public data

This study meets the publication guidelines provided by The Cancer Genome Atlas (TCGA). Gene Expression Profiling Interactive Analysis (GEPIA) was used in this meta-analysis to verify the correlations with OS and DFS and to detect the difference in expression levels of SNHG16 between tumor and normal tissues [35]. The survival analysis was calculated by K–M method and logrank test, and the HRs and *p* value were shown in the figure of K–M curves as previously described [36].

### Statistical analysis

Analyses were conducted by using STATA software (Version 12.0) and Review Manager (RevMan 5.3). Pooled HRs (ORs) and 95% CIs were extracted from the enrolled studies. Chi square-based Q test and *I*^*2*^ statistics were used to determine the heterogeneity across the eligible studies. If *I*^*2*^ > 50% or p-value < 0.05, we considered the heterogeneity was significant and the random-effect model was adopted. On the contrary, the fixed-effect model was applied. Publication bias was evaluated by Egger’s test as well as visual inspection of the symmetry of funnel plot. Sensitivity analysis was performed by sequential omission of each individual study so as to testify the stability of results as previously described [37].

## Results

### Characteristics and eligible studies

**Fig. 1:**
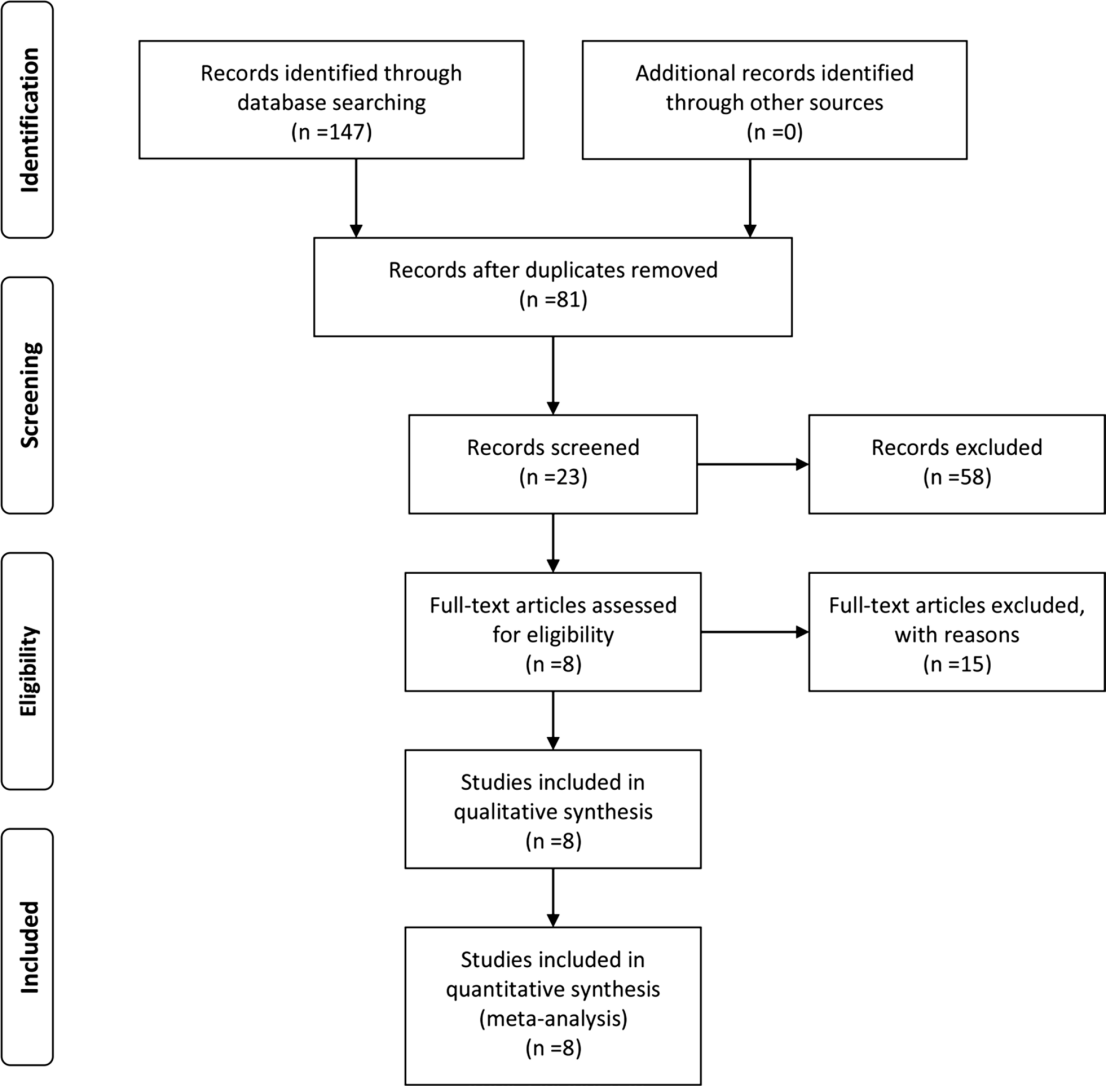
Flow diagram of study selection procedure

A total of 147 publications were initially identified as potential articles, of which 66 were excluded as duplicates. Afterwards, 81 publications were screened via their titles and abstracts, and 58 studies were further excluded since they were case reports, reviews, meeting abstracts, or irrelevant topics. Consequently, 23 full-text articles were thoroughly evaluated. Among them, fifteen studies lacking of survival data were excluded. Thus, eight studies comprising 568 patients were included in the final meta-analysis. The selection procedure was concisely demonstrated by a flow diagram in Fig. 1.

The characteristics of the included studies are presented in Table 1. These articles were published between 2016 and 2019 with the sample size ranging from 38 to 128. All studies were carried out in China and quantitative real-time polymerase chain reaction (qRT-PCR) was applied as the detecting method. Six studies containing five different tumor types utilized tissue samples to detect SNHG16 expression, of which four studies used the median value and the other two studies used the mean value. Association between expression level of SNHG16 with OS were included in the abovementioned studies, including two bladder cancer, one cervical cancer, one esophageal squamous cell carcinoma (ESCC), one glioma, one NSCLC, and one ovarian cancer. The rest two studies used serum samples to detect SNHG16 expression and reported RFS, both of which used the median value as the cut-off value. In all eligible studies, patients were divided into high or low SNHG16 expression groups based on the cut off value. The follow-up months ranged from 60 to 80 months. Univariate and multivariate analysis were both adopted in four studies respectively. As for clinical stage, there were two studies used tumor node metastasis (TNM) classification system, while two studies adopted the International Federation of Gynecology and Obstetrics (FIGO) staging. All of these included studies were cohort studies and of high quality with their NOS scores ≥ 7.

**Table 1:** Summary of the main characteristics of the studies enrolled in the meta-analysis

Study	Year	Country of origin	Tumor type	Sample type	Sample Size	SNHG16 expression		Follow-up months	Detection assay	Clinical stage	Metastasis analysis	Outcome measure	Survival analysis	Cut-off value	Study design	NOS
						High	Low									
Cao et al.	2017	China	Bladder cancer	Tissue	46	25	21	60	qRT-PCR	N/A	LNM/DM	OS	Univariate	Mean	Cohort study	8
Duan et al.	2016	China	Bladder cancer	Serum	59	N/A	N/A	80	qRT-PCR	N/A	LNM	RFS	Univariate	Median	Cohort study	9
Han et al.	2018	China	Esophageal squamous cell carcinoma	Tissue	128	65	63	60	qRT-PCR	TNM I-IV	LNM	OS	Multivariate	Median	Cohort study	8
Han et al.	2019	China	Non-small cell lung cancer	Tissue	66	33	33	60	qRT-PCR	TNM I-IV	LNM	OS/DFS	Multivariate	Median	Cohort study	8
Lu et al.	2018	China	Glioma	Tissue	48	25	23	60	qRT-PCR	N/A	N/A	OS/PFS	Multivariate	Median	Cohort study	8
Yang et al.	2018	China	Ovarian cancer	Tissue	103	47	56	60	qRT-PCR	FIGO I-IV	DM	OS	Univariate	Mean	Cohort study	8
Zhang et al.	2018	China	Bladder cancer	Serum	74	N/A	N/A	76	qRT-PCR	N/A	LNM	RFS	Univariate	Median	Cohort study	9
Zhu et al.	2018	China	Cervical cancer	Tissue	38	22	16	60	qRT-PCR	FIGO I-III	LNM	OS	Multivariate	Median	Cohort study	8

*DFS* disease-free survival, *DM* distant metastasis, *FIGO* the International Federation of Gynecology and Obstetrics, *LNM* lymph node metastasis, *N/A* not available, *NOS* Newcastle–Ottawa Scale, *OS* overall survival, *PFS* progression-free survival, *RFS* recurrence-free survival, *SNHG16* small nucleolar RNA host gene 16, *TNM* tumor node metastasis

### Association between SNHG16 and OS

**Fig. 2:**
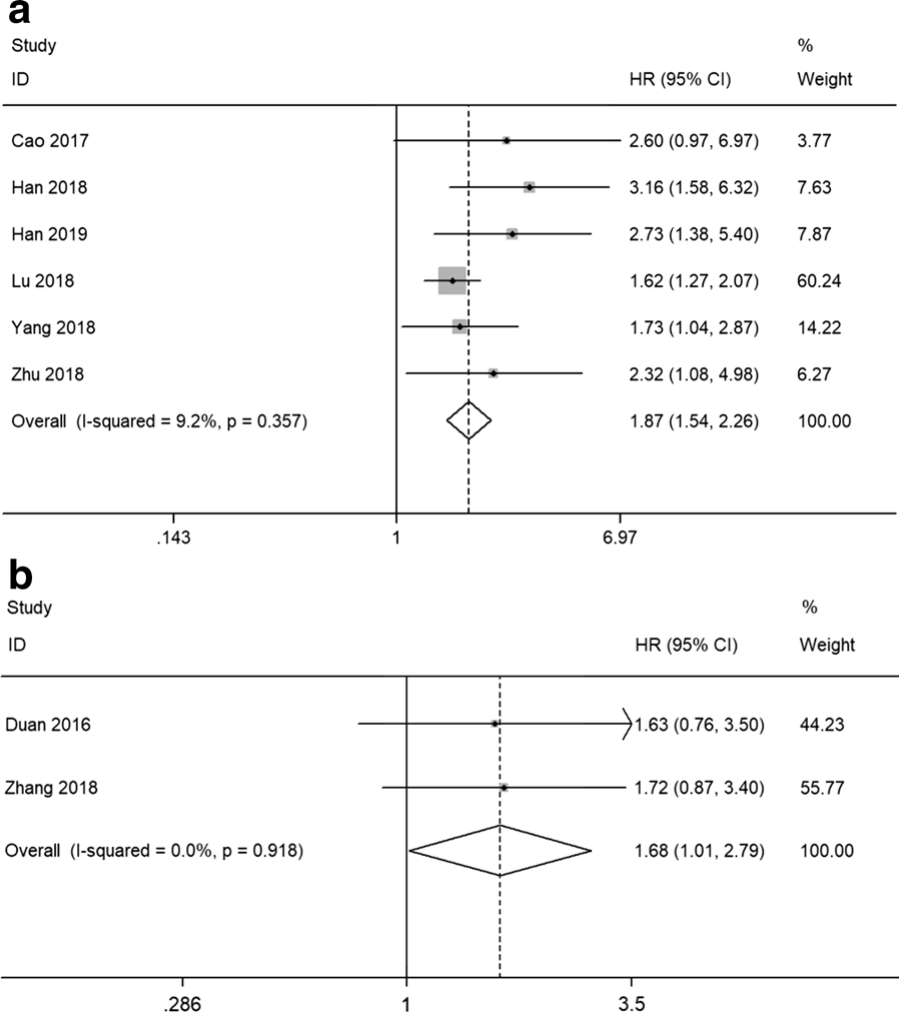
Forest plots of studies evaluating the hazard ratios of high SNHG16 expression in cancers for **a** overall survival and **b** recurrence-free survival

Six studies using tissue samples were included for OS analysis. In the absence of obvious heterogeneity among these studies (*I*^*2*^ = 9.2%, p = 0.357), fixed-effects model was used to calculate the HR and 95% CI. The pooled result demonstrated that high SNHG16 expression significantly associated with worse OS in cancers (HR = 1.87, 95% CI 1.54–2.26, P < 0.001) (Fig. 2a).

### Association between SNHG16 and RFS

Two studies utilizing serum samples provided suitable data for RFS analysis. The fixed-effects model was applied to analyze the pooled HR and its 95% CI since no apparent heterogeneity was observed (*I*^*2*^ = 0.0%, p = 0.918). As shown in Fig. 2b, the results indicated that high SNHG16 expression in serum predicted unfavorable RFS in bladder cancer (HR = 1.68, 95% CI 1.01–2.79, P = 0.045).

### Subgroup analysis of association between SNHG16 and OS

**Fig. 3:**
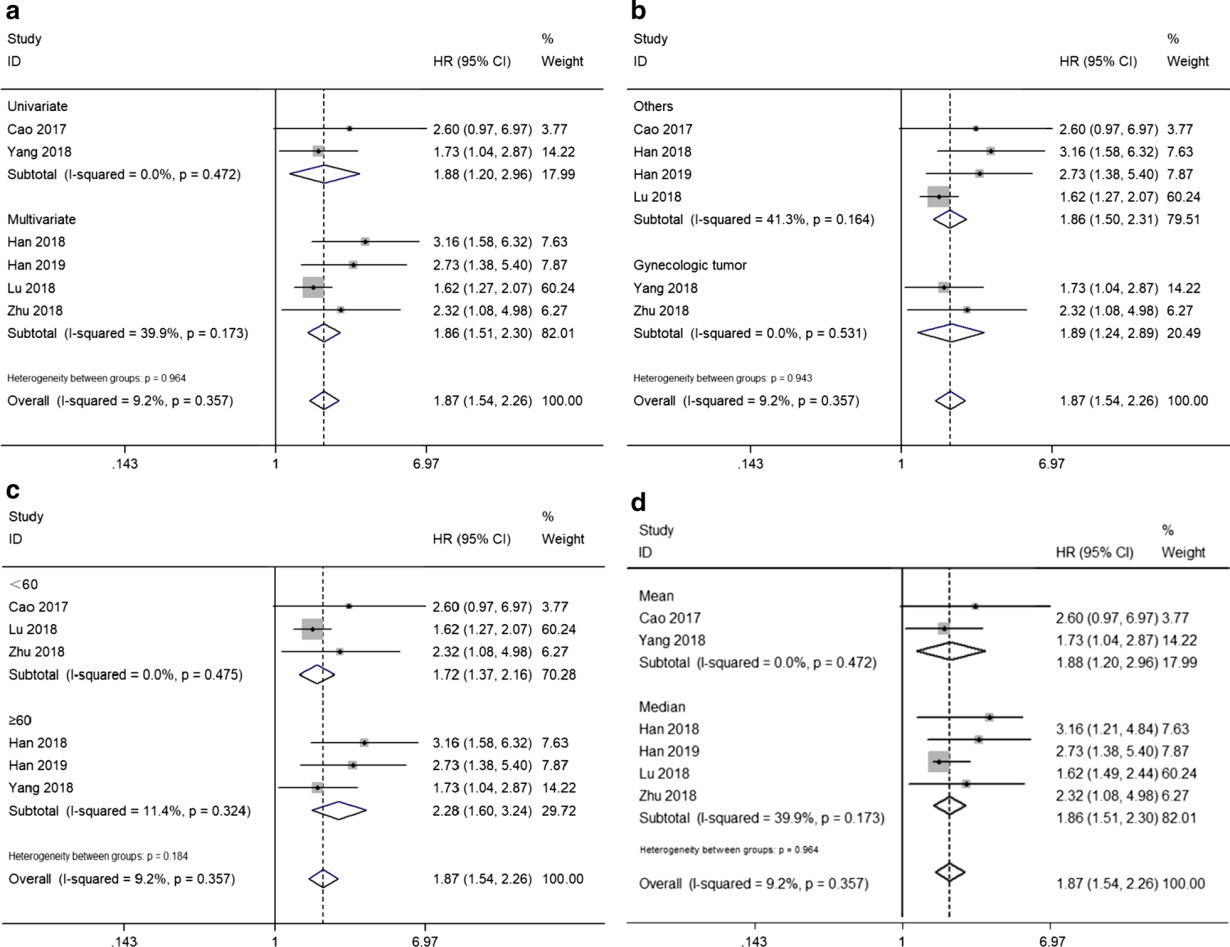
Forest plots evaluating the stratified analyses of SNHG16 expression on OS in regard to subgroup including **a** survival analysis method, **b** tumor type, **c** sample size and **d** cut-off value

Besides, we performed stratified analyses to investigate the association between SNHG16 expression level and OS in divergent subgroups in the light of survival analysis method (univariate or multivariate analysis), tumor type (gynecologic tumor or others), sample size (more or less than 60), and cut-off value (mean or median). As depicted in Fig. 3 and Table 2, all stratified analyses did not alter the predictive value of SNHG16 for OS in several kinds of cancers.

**Table 2:** Stratified analyses of the pooled HRs of overall survival by tumor type, sample size, and survival analysis method

Subgroup analysis	No. of studies	No. of patients	Pooled HR (95% CI)		Heterogeneity	
			Fixed model	p-value	I^2^ (%)	p-value
Survival analysis method						
Univariate	2	149	1.88 (1.20, 2.96)	0.006	0.0	0.472
Multivariate	4	280	1.86 (1.51, 2.30)	< 0.001	39.9	0.173
Tumor type						
Gynecologic tumor	2	141	1.89 (1.24, 2.89)	0.003	0.0	0.531
Others	4	288	1.86 (1.50, 2.31)	< 0.001	41.3	0.164
Sample size						
< 60	3	132	1.72 (1.37, 2.16)	< 0.001	0.0	0.475
≥ 60	3	297	2.28 (1.60, 3.24)	< 0.001	11.4	0.324
Cut-off value						
Mean	2	149	1.88 (1.20, 2.96)	0.006	0.0	0.472
Median	4	280	1.86 (1.51, 2.30)	< 0.001	39.9	0.173

*CI* confidence interval, *HR* hazard ratio

### Association between SNHG16 and clinicopathologic parameters

**Fig. 4:**
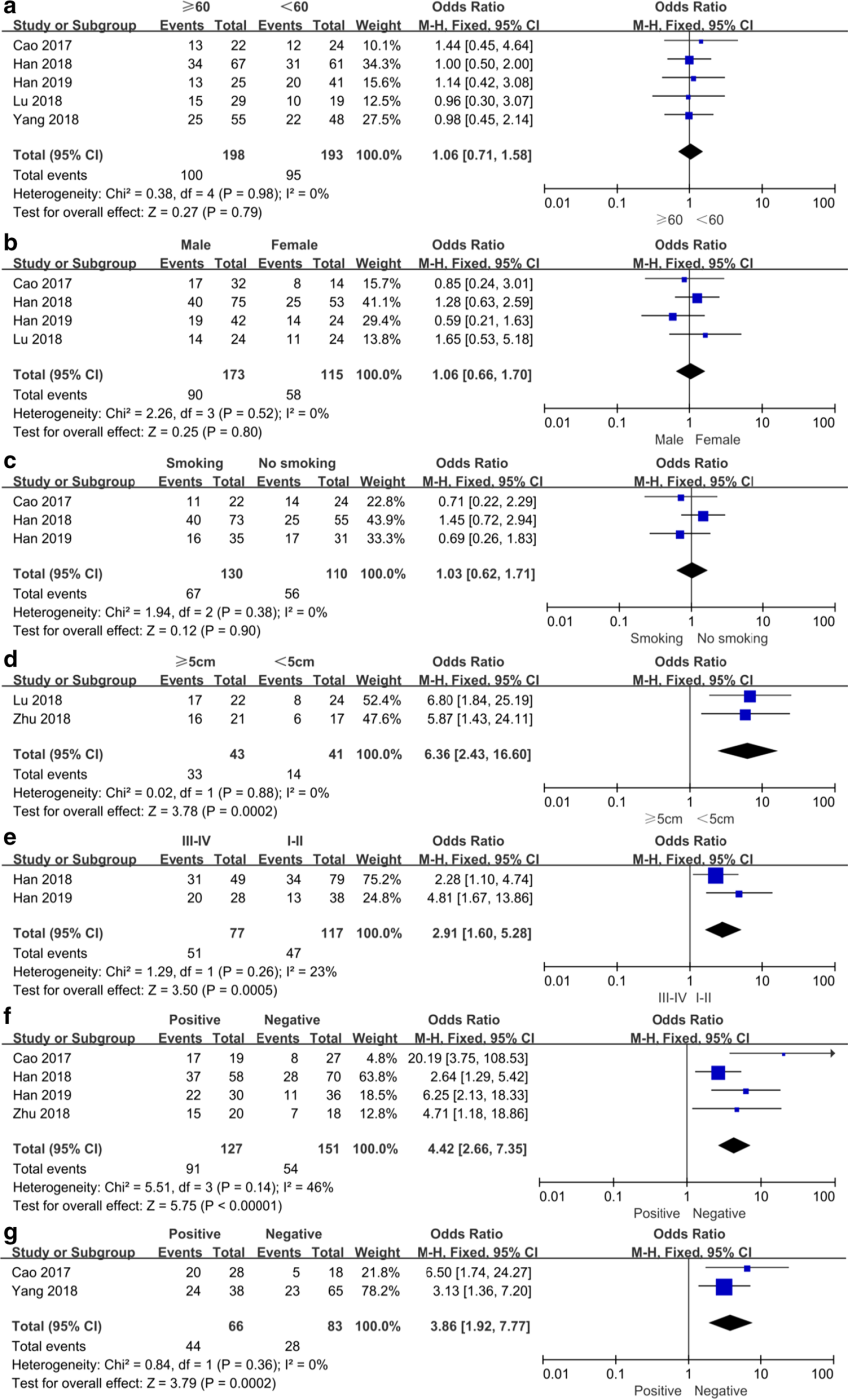
Forest plots evaluating the relationship between SNHG16 expression and clinicopathologic features, including **a** age (≥ 60/< 60), **b** gender, **c** smoking history, **d** tumor size (≥ 5 cm/< 5 cm), **e** clinical stage, **f** lymph node metastasis and **g** distant metastasis

ORs and its 95% CIs were utilized to investigate the correlation between SNHG16 expression level and clinicopathologic parameters including age, gender, smoking history, tumor size, clinical stage, LNM and DM. The results of these analyses were presented in Fig. 4 and Table 3. The fixed-effect model was applied in all analyses. From the pooled ORs, no significant association was detected between SNHG16 expression and age, gender and smoking history. Notably, high SNHG16 expression was significantly correlated with larger tumor size (OR = 6.36, 95% CI 2.43–16.60, P = 0.0002), poor clinical stage (OR = 2.91, 95% CI 1.60–5.28, P = 0.005), LNM (OR = 4.42, 95% CI 2.66–7.35, P = 0.0001) and DM (OR = 3.86, 95% CI 1.92–7.77, P = 0.002).

**Table 3:** Correlation between lncRNA SNHG16 expression and clinicopathologic parameters for cancers

Clinicopathologic parameters	No. of studies	No. of participants	Pooled OR (95% CI)	P	Model	Heterogeneity
						Chi^2^, P-value, I^2^ (%)
Age (≥ 60/< 60)	5	391	1.06 (0.71, 1.58)	0.79	Fixed	0.38, 0.98, 0
Gender	4	288	1.06 (0.66, 1.70)	0.80	Fixed	2.26, 0.52, 0
Smoking history	3	240	1.03 (0.62, 1.71)	0.90	Fixed	1.94, 0.38, 0
Tumor size (≥ 5 cm/<5 cm)	2	84	6.36 (2.43, 16.60)	0.0002	Fixed	0.02, 0.88, 0
Clinical stage	2	194	2.91 (1.60, 5.28)	0.0005	Fixed	1.29, 0.26, 23
LNM	4	278	4.42 (2.66, 7.35)	< 0.00001	Fixed	5.51, 0.14, 46
DM	2	149	3.86 (1.92, 7.77)	0.0002	Fixed	0.84, 0.36, 0

*CI* confidence interval, *DM* distant metastasis, *LNM* lymph node metastasis, *OR* odds ratio, *SNHG16* small nucleolar RNA host gene 16

### Sensitivity analysis

**Fig. 5:**
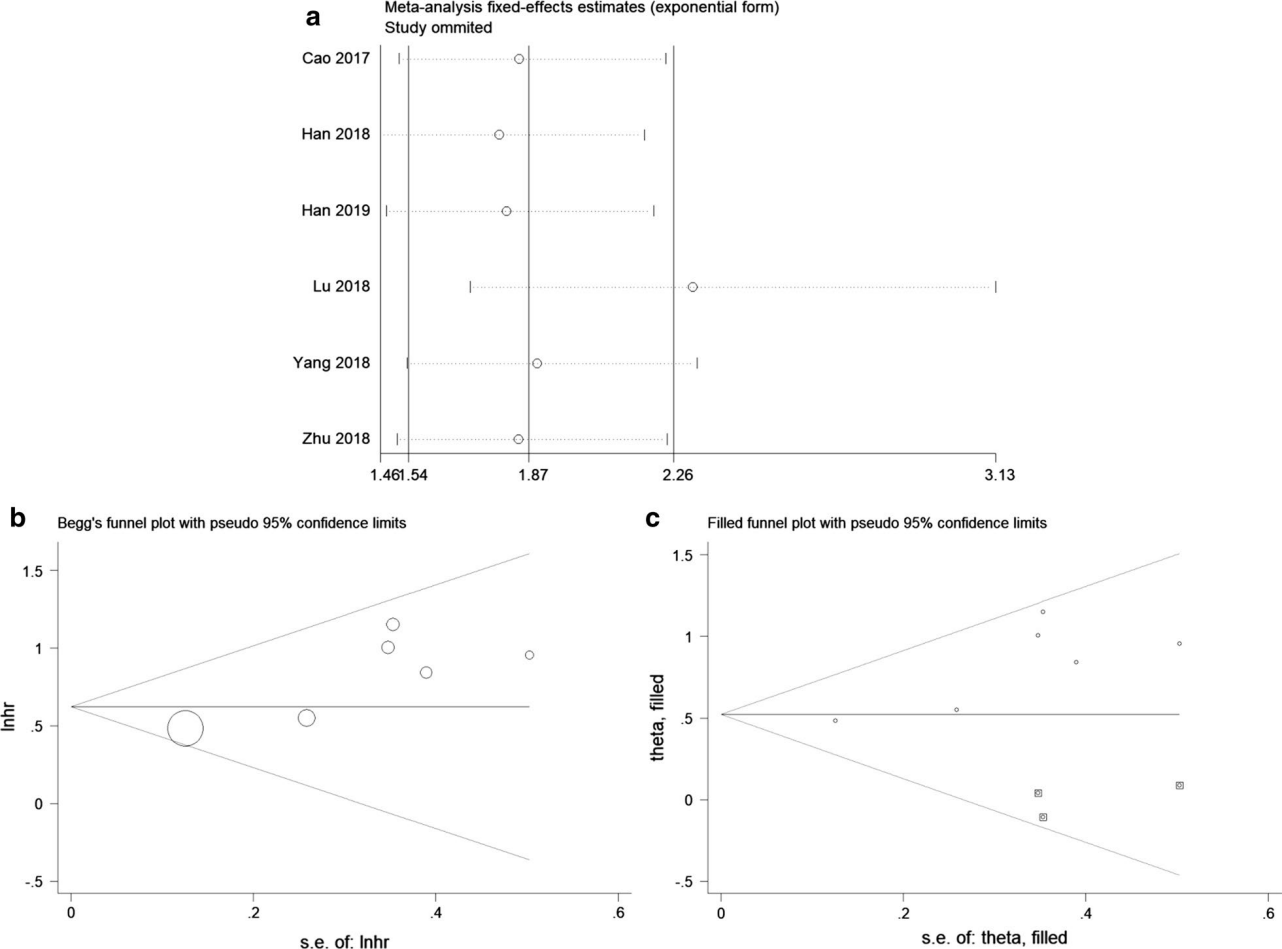
**a** Sensitivity analysis of pooled HR for overall survival. **b** Begg’s funnel plot of SNHG16 for overall survival. **c** Funnel plot of “Trim and Fill analysis” (The trim and fill adjusted HR = 1.69, 95% CI 1.41–2.01, fixed-effects model)

In order to test the stability of the pooled result of the association between SNHG16 expression and OS, sensitivity analysis was conducted by removing each eligible study. As demonstrated in Fig. 5a, when “Lu 2018” [21] was removed, the pooled result fluctuated. Subsequently, the pooled HR was calculated again after removing “Lu 2018”, and the result showed that high SNHG16 expression still predicted worse OS in multiple cancers (HR = 2.31, 95% CI 1.71–3.13, P < 0.001), which meant that the significance of the pooled result was not altered by the influential study. Therefore, our pooled result of SNHG16 expression on prediction of OS was reliable.

### Publication bias

For meta-analysis of the association between SNHG16 expression and OS, Begg’s funnel plot and Egger’s regression test were performed to test for publication bias. There was evidence of publication bias based on asymmetry in the Begg’s funnel plot (Fig. 5b) as well as the result of Egger’s regression test (P = 0.025). Later, “Trim and Fill analysis” was adopted to evaluate the influence of publication bias as previously described [38]. As depicted in Fig. 5c, the adjusted HR and 95% CI was 1.69 (1.41–2.01), indicating that the publication bias did not have significant influence on the pooled result, and thus our result was credible.

### Validation of the results in TCGA dataset

**Fig. 6:**
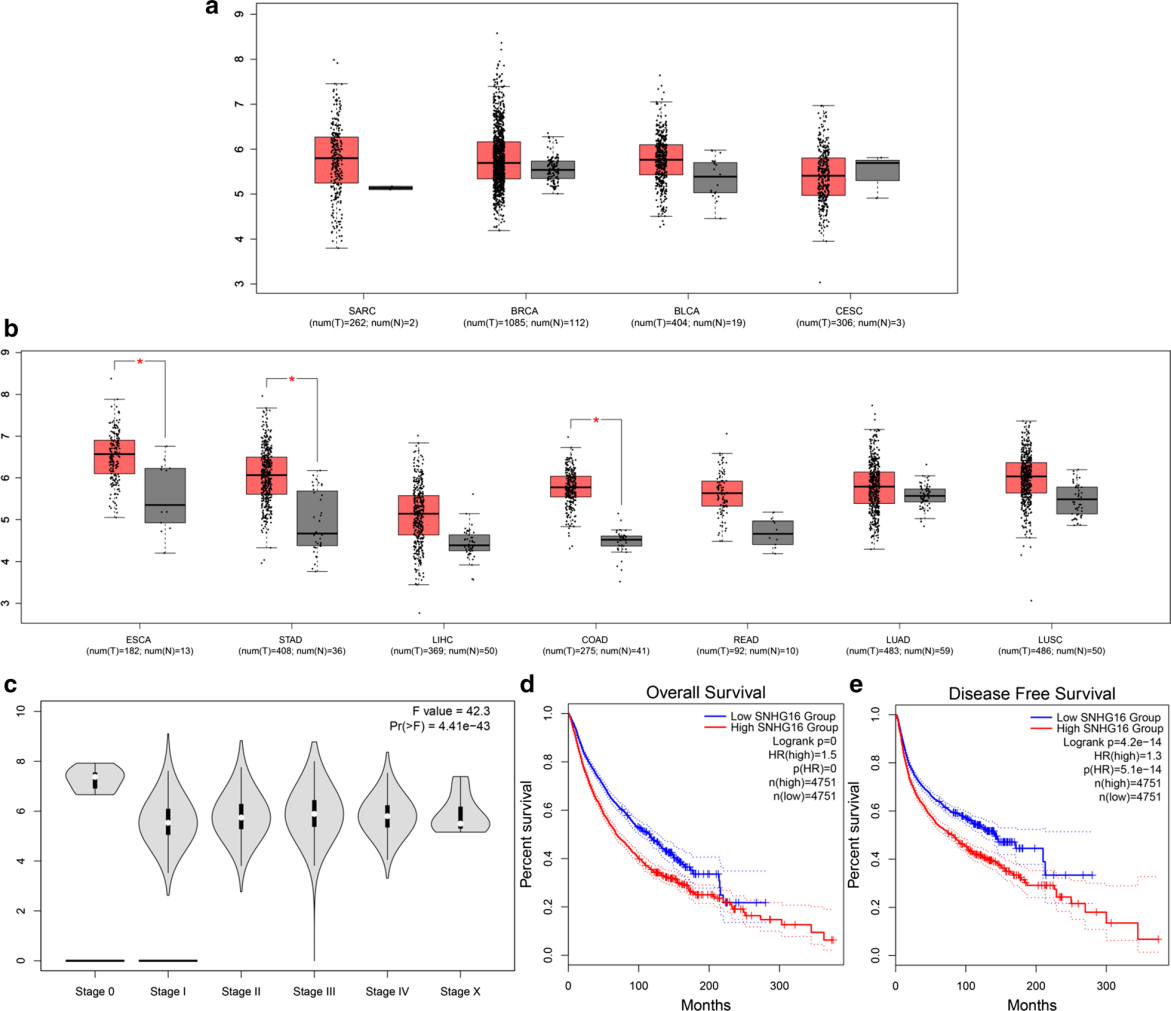
Validation of SNHG16 expression in various cancers in TCGA cohort. **a** The expression levels of SNHG16 in SARC (sarcoma), BRCA (breast invasion carcinoma), BLCA (bladder urothelial carcinoma), and CESC (cervical squamous cell carcinoma and endocervical adenocarcinoma). **b** The expression levels of SNHG16 in ESCA (esophageal carcinoma), STAD (stomach adenocarcinoma), LIHC (liver hepatocellular carcinoma), COAD (colon adenocarcinoma), READ (rectum adenocarcinoma), LUAD (lung adenocarcinoma), and LUSC (lung squamous cell carcinoma). **c** Violin plot showing SNHG16 expression in different major clinical stage of pan-cancers in TCGA cohort. **d** Overall survival plot of SNHG16 in TCGA cohort (n = 9502). **e** Disease-free survival plot of SNHG16 in TCGA cohort (n = 9502)

In order to further validate our results, we utilized TCGA dataset to investigate SNHG16 expression level in various cancers. As shown in Fig. 6, SNHG16 was aberrantly expressed in sarcoma, breast invasion carcinoma, bladder urothelial carcinoma, esophageal carcinoma, stomach adenocarcinoma, liver hepatocellular carcinoma, colon adenocarcinoma, rectum adenocarcinoma, lung adenocarcinoma, and lung squamous cell carcinoma when compared with normal control. In addition, the violin plot showed that SNHG16 expression level was significantly associated with clinical stage in human pan-cancers. Moreover, we adopted survival plots in GEPIA via merging SNHG16 expression data and OS (DFS) data of malignancies from all of the TCGA dataset, which containing 9502 patients divided into high or low expression group based on SNHG16 expression. The results indicated that the upregulated SNHG16 expression predicted unfavorable OS and DFS, which certified our results in this meta-analysis.

## Discussion

LncRNAs were previously recognized as “junk DNA” or “transcriptional noise” and did not attract too much attention among researchers in the past decades [39]. Recently, role of lncRNAs in human diseases has grown in importance due to advancement in the application of next-generation genome wide sequencing and microarray [40–43]. Previous studies have demonstrated that lncRNAs are abnormally expressed in cancer and acted as oncogenes or tumor suppressors, revealing an important role in cancer prognosis [44–46]. For instance, tubulin alpha 4b (TUBA4B) was significantly decreased in cancer tissues compared with adjacent normal specimens [47]. Low TUBA4B expression was closely correlated with pathological grade, LNM, OS, DFS, and RFS in cancer patients and can be a novel biomarker for the prognosis of various cancers [47, 48]. Likewise, plasmacytoma variant translocation gene 1 (PVT1) expressions in cancer tissues were higher than paired healthy controls. Overexpression of serum PVT1 was markedly associated with larger tumor size, advanced clinical stage, and accurately predicted the disease and poor prognosis [49–51]. Moreover, PVT1 could engage in multiple signaling pathways or act as competitive endogenous RNA (ceRNA) to affect the biological function of cancer cells via interacting with miRNAs and target genes, indicating a novel perspective for therapeutic strategies of human cancers [51, 52].

SNHG16 is a newly identified lncRNA and has been reported to be aberrantly expressed in multiple malignancies. For instance, SNHG16 expression levels were significantly upregulated in osteosarcoma [15], glioma [20, 21], colorectal adenocarcinoma [27], breast cancer [23], cervical cancer [26], ovarian cancer [24], bladder cancer [17, 18], ESCC [19, 28], NSCLC [11], oral squamous cell carcinoma [13], and acute lymphoblastic leukemia [53], but downregulated in HCC [22]. However, data in TCGA showed that SNHG16 was overexpressed in liver hepatocellular carcinoma (LIHC), which is contradictory to the study reported by Xu et al. [22]. Additionally, a strong correlation between SNHG16 and cancer biological functions has been well illustrated in the literature. Overexpression of SNHG16 could lead to changes in cancer cell proliferation [18, 19, 24], apoptosis [21], migration [23], invasion [11, 26], epithelial-mesenchymal transition (EMT) [19] and chemoresistance in a majority of cancers [22]. In our meta-analysis, we explored the correlation between SNHG16 expression levels and cancer prognostic parameters. The pooled results revealed that high expression levels of SNHG16 predicted unfavorable OS. Furthermore, a shorter RFS was observed in bladder cancer patients with high expression of SNHG16 in serum, implying that expression of SNHG16 in serum was a hazardous factor for the recurrence of bladder cancer. Besides, SNHG16 was also correlated with PFS and DFS in two studies. Glioma patients with higher SNHG16 expression had a significantly poorer PFS based on the result of multivariate analysis (HR = 3.167, 95% CI 1.552–6.231) [21]. SNHG16 expression could serve as independent predictor for DFS in NSCLC patients (HR = 2.641, 95% CI 1.394–5.002) [11]. Therefore, SNHG16 upregulation was closely associated with poor prognosis. Our pooled results also showed that patients with high SNHG16 expression were more prone to worse clinicopathological features including larger tumor size, advanced clinical tumor stage, LNM and DM. Besides, there were other parameters that cannot be included in the meta-analysis since they were only reported in a single study. For instance, cervical patients with high SNHG16 expression had poorer differentiation (P = 0.047) and worse FIGO stage (P = 0.008). In ovarian cancer, higher SNHG16 expression predicted higher histological grade (P = 0.002) [24]. Moreover, subgroup analyses showed that survival analysis method, tumor type, sample size and cut-off value did not alter the predictive value of SNHG16 on OS. Taken together, SNHG16 could serve as a functional regulator and potential biomarker of poor prognosis in pan-cancer patients.

**Fig. 7:**
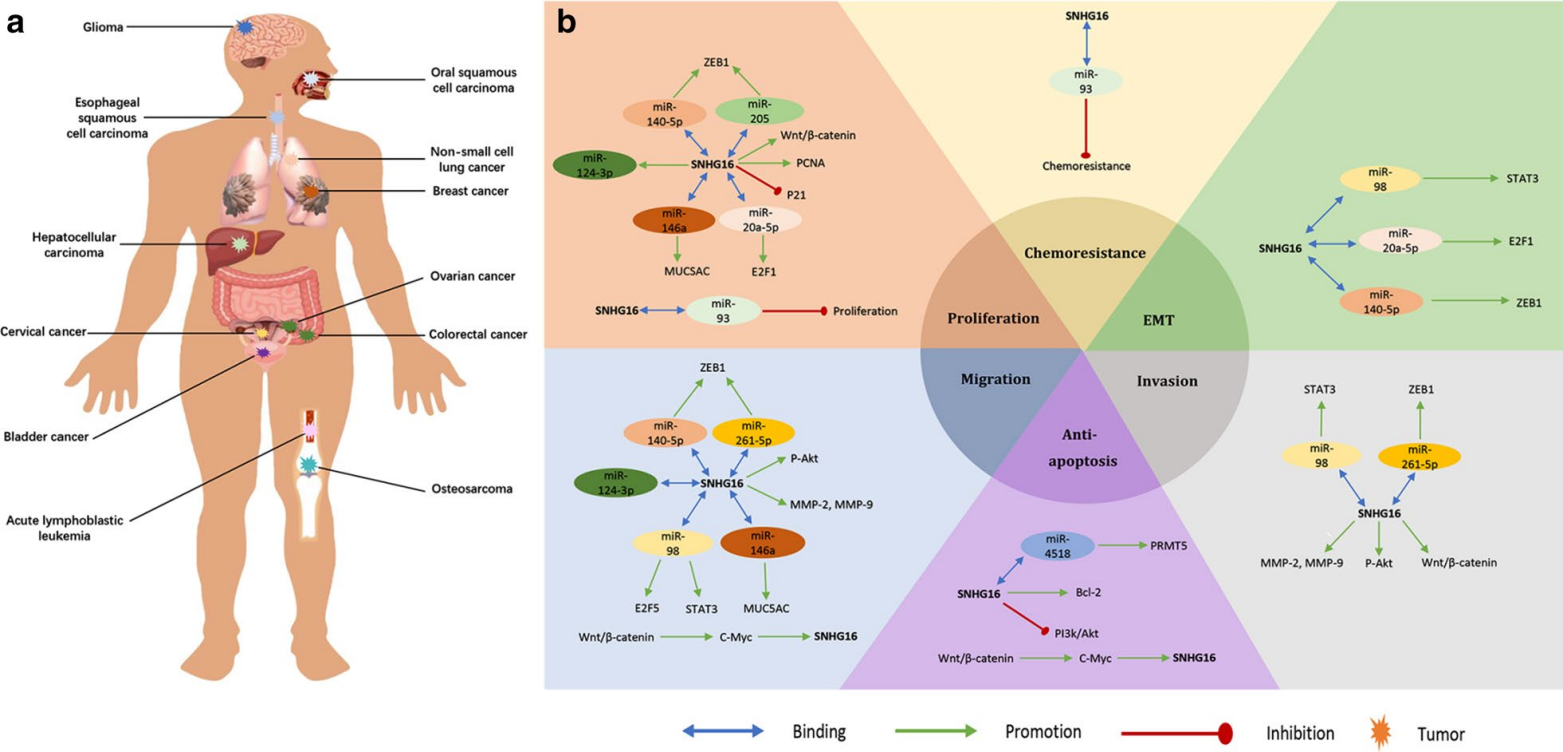
Summary of aberrant expression of SNHG16 in various types of human malignancies (**a**). A comprehensive biological role of SNHG16 in carcinogenesis of human cancers, including proliferation, apoptosis, migration, invasion, epithelial-mesenchymal transition (EMT) and chemoresistance regulation. SNHG16 may function as competitive endogenous RNA (ceRNA) by directly sponge to miRNA and subsequently regulating target geness, or interact with several signaling pathways in the pathogenesis of cancers (**b**)

Further mechanism studies highlighted that SNHG16 may function as ceRNA by directly sponge to miRNA and thereby regulating target genes in cancers, such as miR-205/ZEB1 in osteosarcoma [15], hsa-miR-93 in HCC [22], miR-4518/PRMT5 or miR-20a-5p/E2F1 in glioma [20, 21], miR-146a/MUC5AC in NSCLC [11], miR-98/E2F5 in breast cancer [23], miR-216A-5p/ZEB1 in cervical cancer [26], miR-140-5p/ZEB1 in ESCC [19], miR-98/STAT3 in bladder cancer [17], and hsa-miR-124-3p in acute lymphoblastic leukemia [53]. Besides, SNHG16 could interact with a variety of signaling pathways including Wnt/β-catenin [17, 27] and PI3 K/Akt [21, 24] in the pathogenesis of cancers. A brief summary of SNHG16 with their potential targets, functional roles, signaling pathways, and relevant miRNAs was presented in Table 4 and illustrated in Fig. 7.

**Table 4:** Summary of lncRNA NNT-AS1 with their potential targets, functional roles, signaling pathways, and related miRNAs

Tumor type	Potential targets	Functional roles	Pathways	Related miRNAs
Acute lymphoblastic leukemia	–	Cell proliferation and migration		miR-124-3p
Bladder cancer	STAT3	Cell invasion, migration and EMT	–	miR-98
Bladder cancer	P21	Cell proliferation	–	–
Breast cancer	E2F5	Cell migration	–	miR-98
Cervical cancer	ZEB1	Cell invasion and migration	–	miR-216-5p
Colorectal cancer		Cell migration and anti-apoptosis	Wnt/β-catenin	–
Esophageal squamous cell carcinoma	ZEB1	Cell proliferation, migration and EMT	–	MiR-140-5P
Esophageal squamous cell carcinoma	–	Cell proliferation	Wnt/β-catenin	–
Glioma	PRMT5, Bcl-2	Anti-apoptosis	PI3 K/Akt	miR-4518
Glioma	E2F1	Cell proliferation and EMT	–	miR-20a-5p
Hepatocellular carcinoma	–	Anti-proliferation and anti-chemoresistance	–	miR-93
Non-small cell lung cancer	MUC5AC	Cell proliferation and migration	–	miR-146a
Osteosarcoma	ZEB1	Cell proliferation	–	miR-205
Oral squamous cell carcinoma	PCNA, MMP-2, MMP-9	Cell proliferation, invasion and migration	–	–
Ovarian cancer	p-Akt, MMP-9	Cell invasion and migration	–	–

*Akt* protein kinase B, *Bcl*-*2* B-cell lymphoma 2, *E2F1* E2F transcription factor 1, *E2F5* E2F transcription factor 5, *EMT* epithelial–mesenchymal transition, *MMP*-*2* matrix metalloproteinase 2, *MMP*-*9* matrix metalloproteinase 9, *MUC5AC* mucin 5AC, *PI3* *K* phosphoinositide 3-kinase, *PCNA* proliferating cell nuclear antigen, *p*-*Akt* phosphorylated protein kinase B, *PRMT5* protein arginine methyltransferase 5, *STAT3* signal transducer and activator of transcription 3, *ZEB1* zinc finger E-box-binding homeobox 1

Of note, several limitations should be addressed in current meta-analysis. First, all the included studies were performed within Chinese populations, thus caution must be applied, as the findings might not be able to generalize to other populations. Second, some of the HR values had been computed through reconstruction of K–M curves instead of directly obtaining from the original studies, which inevitably could generate possible bias. Third, all included studies set the cut-off value as mean or median value without detailed description on the calculation process or providing original data. Therefore, the real cut-off value for each study was unknown, and the different cut-off values across the selected studies may lead to potential bias. Fourth, heterogeneity may exist in different treatments for diverse cancer patients, which may contribute to the bias. Fifth, most included articles reported positive results rather than negative results, which may introduce publication bias.

## Conclusions

In conclusion, our results infer that high SNHG16 expression was strongly associated with unfavorable survival outcome of several cancers and therefore might serve as a novel prognostic biomarker and potential therapeutic target in cancer patients. However, studies with a larger sample size on the current topic are still needed to substantiate these results.

## Data Availability

The datasets used and/or analyzed during the current study are available from the corresponding author on reasonable request.

## Abbreviations

Akt: protein kinase B
ceRNA: competitive endogenous RNA
CI: confidence interval
CNKI: China National Knowledge Infrastructure
DFS: disease-free survival
DM: distant metastasis
E2F1: E2F transcription factor 1
E2F5: E2F transcription factor 5
EMT: epithelial–mesenchymal transition
ESCC: esophageal squamous cell carcinoma
FIGO: the International Federation of Gynecology and Obstetrics
GEPIA: Gene Expression Profiling Interactive Analysis
HCC: hepatocellular carcinoma
HR: hazard ratio
JAK2: Janus kinase 2
K–M curve: Kaplan–Meier curve
LNM: lymph node metastasis
MMP-2/-9: matrix metalloproteinase 2/9
MUC5AC: mucin 5AC
NOS: Newcastle–Ottawa Scale
NSCLC: non-small cell lung cancer
OR: odd ratio
OS: overall survival
PFS: progression-free survival
PI3 K: phosphoinositide 3-kinase
PRMT5: protein arginine methyltransferase 5
PVT1: plasmacytoma variant translocation gene 1
qRT-PCR: quantitative real-time polymerase chain reaction
RFS: recurrence-free survival
SNHG16: small nucleolar RNA host gene 16
STAT3: signal transducer and activator of transcription 3
TCGA: The Cancer Genome Atlas
TNM: tumor node metastasis
TUBA4B: tubulin alpha 4b
ZEB1: zinc finger E-box-binding homeobox 1
